# A Detailed Analysis of the Past 20 Years of US FDA-Approved Prescription to Over-the-Counter Switches

**DOI:** 10.1007/s43441-023-00547-9

**Published:** 2023-06-25

**Authors:** Matt Fisher, Kapil Rawal

**Affiliations:** Haleon, 184 Liberty Corner Rd, Warren, NJ 07059-6796 USA

**Keywords:** FDA, Over-the-counter, Nonprescription, Rx-to-OTC switch, Consumer healthcare

## Abstract

**Objectives:**

This evaluation assesses the quantity, uniqueness, and innovative nature of the past 20 years of Rx-to-OTC (RTO) switches, where a current prescription (Rx) product is reclassified for over-the-counter (OTC) status. Broadening access to more OTC drugs with well-established safety and efficacy could help to reduce healthcare expenditure and address public health challenges.

**Methods:**

The FDA-maintained RTO switch list website was accessed to generate the primary dataset. Each product listed was assessed for the current OTC availability in the United States of its active ingredient, pharmacological class, and indication to determine its innovative quality. Descriptive statistics were employed in this study.

**Results:**

From January 2002 through August 2022 there were 45 RTO switches. Among these, 51.1% involved a new to OTC active ingredient, 22.2% involved a new pharmacological class, 6.6% involved a new indication, and 82.2% were considered follow-on products that introduced a new to OTC active ingredient or new dosage form of an already marketed active ingredient to treat an existing OTC indication. A small minority (6.6%) were considered an exceptional innovation that would offer US consumers a genuinely novel OTC product, providing a new to OTC active pharmaceutical ingredient, pharmacological class, and indication. Overall, there was 1 exceptional innovation every 6.7 years.

**Conclusions:**

Over 40 RTO switches have come to the OTC market in the past 20 years; however, exceptional innovations that expand access to new to OTC active ingredients for new indications are rare. Policies and strategies that result in more innovative switches that can benefit consumers and public health should be evaluated.

**Supplementary Information:**

The online version contains supplementary material available at 10.1007/s43441-023-00547-9.

## Introduction

RTO switches occur when the FDA approves a sponsor’s new drug application or supplemental application for OTC status of a current Rx product. An RTO switch can manifest in two distinct forms: as a full RTO switch or as a partial RTO switch. A full RTO switch is when the current Rx-only NDA is switched to OTC status in its entirety, with no prescription availability remaining post-switch. A partial RTO switch is when a sponsor seeks to convert only some conditions of use (such as a specific dose or indication) to OTC status. This type of RTO switch requires the sponsor to submit a new NDA. Both types of RTO switches require a data package outlining the safety and efficacy for use as an OTC product. This assists the FDA in determining the risk–benefit of switching the prescription product to OTC status. The data package may include data from existing or new randomized controlled trials as well as data and information associated with the post-marketing safety experience. In addition to information related to safety and efficacy, the sponsor is often required to conduct consumer label comprehension studies and self-selection studies [1–3]. Label comprehension studies usually involve the recruitment of participants that reflect the general population to review a proposed OTC label and answer questions to determine whether the label can be sufficiently understood. Self-selection studies involve participants that would be consistent with the proposed product’s target population to determine whether respondents can adequately assess whether or not the product is appropriate for themselves.

Certain RTO switch programs will include an actual use study, which is an evaluation of the proposed product under simulated real world OTC conditions, without the supervision of a healthcare provider. Following the NDA submission by the sponsor, the FDA may choose to convene a non-prescription drug advisory committee meeting to seek input from the public as well as independent advisors on various aspects of the proposed RTO switch [4].

RTO switches offer the opportunity to continue disease awareness and education while greatly expanding access to safe and effective medications that are often at a lower cost and greater convenience than when only available by prescription [5, 6]. The need for innovative OTC options is critical, particularly with existing inequities in the US healthcare system [7], coupled with an increasing population of informed consumers with greater access to education about medical conditions and medications [8–11]. Broadening access to more OTC drugs with well-established safety and efficacy could help to address public health challenges including cost concerns, shortage of physicians, accessibility, healthcare inequality, lost productivity, and undertreatment of certain conditions [6, 12–15]. Accessible OTC treatment options can also help alleviate burden on the healthcare system, allowing patients to self-treat. Greater patient and consumer autonomy in healthcare can empower a more health-conscious and informed population. The objective of this research was to quantify the innovative nature of the past 20 years of RTO switches that have been FDA-approved in the US (2002–2022).

## Materials and Methods

The FDA-maintained RTO switch list website was accessed to generate the primary dataset [16]. Each product listed was assessed for its active ingredient, pharmacological class, and indication to determine its innovative qualities. Innovative qualities were assessed in relation to whether the active ingredient, pharmacological class, or indication were previously available OTC in the United States. A “new to OTC active ingredient” was defined as a novel active pharmaceutical ingredient that was not previously available in a marketed OTC product. A “follow-on product” was defined as a new dosage form of an already marketed active ingredient or a new product that offers a new to OTC active ingredient within a pharmacological class and indication that already exists on the OTC market. These are sometimes also referred to as “me-too” products. A “new pharmacological class” was defined as a product within a class of drugs that was not previously available to consumers in a marketed OTC product prior to the switch. A “new indication” was defined as a product that introduced a novel OTC indication that was not previously FDA approved for consumers. An “exceptional innovation” was defined as one that introduced a new to OTC active pharmaceutical ingredient, pharmacological class, and indication in the same switch of which none were previously available in the OTC marketplace. Descriptive statistics were employed in this study.

## Results

From January 2002 through August 2022 there were 45 RTO switches. Over this period, a significant majority (82.2%) of switches were follow-on products. Just over half (51.1%) involved a new to OTC active pharmaceutical ingredient with less than one-fourth (22.2%) providing a new pharmacological class of medications. Three switches (6.6%) were considered exceptional innovations (Table 1). As such, for every one exceptional innovation, there were 12 follow-on products. On average, during the study period, there was one exceptional innovation RTO switch every 6.7 years.

**Table 1:** 20 years of OTC switches

Category	2002–2022 (*n* = 45)
New to OTC active ingredient	23 (51.1%)
New pharmacological class	10 (22.2%)
New indication	3 (6.6%)
Exceptional innovation	3 (6.6%)
Follow-on product	37 (82.2%)

Values are *n* (%); “Exceptional innovation” refers to a new product that introduces a new to OTC active ingredient, pharmacological class, and indication to the OTC marketplace; “Follow-on product” is defined as a new product that only offers a new to OTC active ingredient or a new dosage form of an already marketed active ingredient; however, the pharmacological class and indication already exist on the OTC market; “New to OTC active ingredient” is a novel active pharmaceutical ingredient that was not previously available in an OTC product; “New indication” refers to a product that introduced a novel OTC indication that was not previously available for consumers; “New pharmacological class” was defined as a product within a class of drugs that was not previously available to consumers OTC prior to the switch

**Fig. 1:**
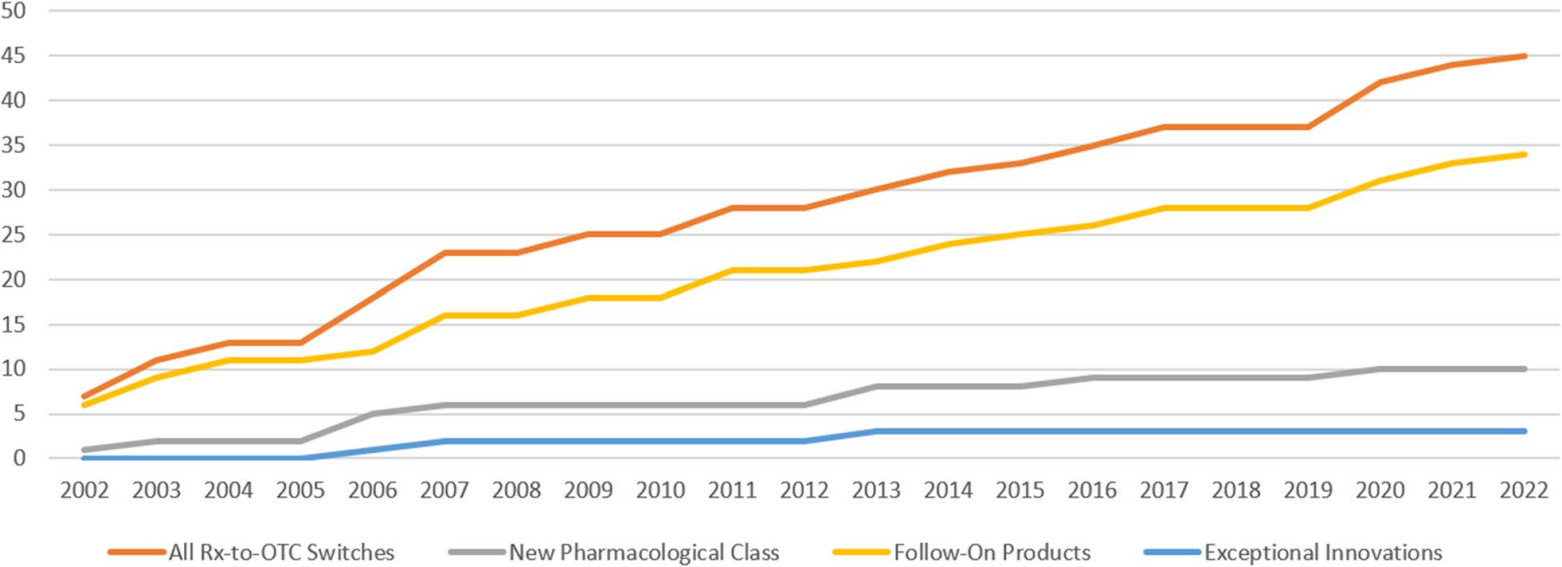
20 years of OTC switches

Figure 1 displays the switches over time demonstrating the increasing number of switches that are simply follow-on products (Fig. 1). Products that are predominantly for seasonal allergies (29/45) or for gastrointestinal symptoms (6/45) form a large proportion (64 and 13%, respectively) of switches in the observation period (Supplemental Material). Out of the nine new pharmacological classes switched during the last 20 years, only 4 (44.4%) primarily function via the systemic route (Supplemental Material).

Three exceptional products were identified over the entire observation period (Table 2). These RTO switches include levonorgestrel for emergency contraception, orlistat for weight loss, and oxybutynin for overactive bladder in women.

**Table 2:** Exceptional innovations in OTC switch

Brand	Generic	Year approved for RTO switch	Sponsor company (at the time of approval)	Indication	Description
Plan B	Levonorgestrel	2006	Duramed Pharmaceuticals	Emergency Contraceptive	− Single tablet taken by mouth within 72 h of unprotected intercourse − Does not negatively affect chances of future pregnancies − Does not harm an existing pregnancy − Does not interfere with regular birth control methods − Does not protect against sexually transmitted diseases − Cost: $40–50 USD
Alli	Orlistat	2007	GSK Consumer Healthcare	Weight Loss	− Only FDA-approved OTC weight loss aid − Can help lose 50% or more weight than dieting alone when used as directed − 10–15% visceral fat loss after 24 weeks of use − Still requires regular exercise, three balanced meals (reduced-calorie, low-fat), and plenty of water − May reduce absorption of fat-soluble vitamins − Cost: $50–70 USD
Oxytrol	Oxybutynin	2013	Merck Consumer Care	Overactive Bladder	− First FDA-approved OTC treatment for overactive bladder − Easy to administer patch applied to the hip, abdomen, or buttock − Can be worn during everyday activities, such as showering or exercising − Must wear for 3–4 days − Cost: $15–30 USD

## Discussion

The main results from this analysis demonstrate that there are two or three RTO switches per year, on average. Exceptional innovations that offer novel active ingredients for unprecedented indications are extremely rare. To our knowledge this is the first analysis of this kind.

Simply viewing the RTO switches during the past 20 years leads one to observe that the majority of new pharmacological class switches (5 out of 9) work via the non-systemic route (Supplemental Material). These agents (polyethylene glycol 3350, orlistat, triamcinolone acetonide, adapalene, and ivermectin) do not achieve appreciable systemic absorption and their action is limited to a certain function or area of the body. The four remaining agents work systemically (Supplemental Material). However, it is important to note that the last systemically acting new pharmacological class to enter the market as a switch was a competitive antagonist for acetylcholine in the form of transdermal oxybutynin in 2013, nearly 10 years ago (Supplemental Material). It is unclear what has led to this relatively long absence of systemically active RTO switches; however, the underlying reasons should be investigated. This is where open dialogue and debate can play a role to support products that can offer potential opportunities to address important therapeutic gaps.

Over the observation period, it appears as if many new to OTC active ingredients are entering the market as switches, but looking more closely at the data shows that an overwhelming majority are follow-on products, not innovative products. The influx of new to OTC active ingredients as follow-on products within the same pharmacologic class may, however, increase competition, allow for broader consumer choices, and result in lower prices for consumers. Ultimately, the underlying issues that limit exceptional innovations in the OTC drug marketplace remain unclear.

Certainly, as indications for potential OTC drugs become more complex, confirming safe and effective use by consumers may be challenging. Despite theoretical concerns, there remains several possible opportunities for novel OTC drugs.

OTC statins (HMG-CoA reductase inhibitors) have long been discussed as an opportunity to address undertreatment of elevated cholesterol and for the reduction of vascular events including myocardial infarctions and strokes [17, 18]. Despite the potential for substantial benefits, several issues raised by certain stakeholders have impeded the opportunity associated with OTC statins. These include challenges with appropriate self-diagnosis, as well possible overtreatment or undertreatment of the condition. A new approach to bringing rosuvastatin directly to consumers is being investigated, although the consumer experience is unclear and appears to break from traditional means of acquiring OTC drugs [19, 20]. This approach incorporates technology, specifically a self-accessed web-based application on an internet capable device, to qualify a consumer for purchasing rosuvastatin based on their responses [19, 20]. This deviates from the traditional retail approach to accessing OTC drugs. Adding barriers to accessing OTC drugs that can otherwise be used safely and effectively under usual conditions, such as making the product e-commerce only, limiting distribution to certain retail establishments, requiring mobile application downloads, incorporating lengthy questionnaires, or requiring to add detailed biometric data (such as blood pressure), introduces some of the same challenges that are present for prescription products and would do little to address the undertreatment and lack of adherence concerns.

In addition to statins, other novel OTC drugs can and should be seriously considered, as described further below. Recently, nonprescription oral contraceptives have come into focus [21]. These products are undergoing the required testing for OTC use, however, given the important differences between progesterone-only pills and combination estrogen-progesterone options, it appears that the latter have more challenges related to self-selection, such as blood pressure and smoking warnings as well as thromboembolic concerns. [22]. Again, barriers that limit progress for bringing novel OTC switches to the market should be justifiable rather than simply based on theoretical concerns. Furthermore, potential safety concerns as well as product benefits can be examined in clinical studies that simulate a naturalistic OTC environment to assess whether potential or theoretical issues would sufficiently undermine the benefits of the product [23].

Prior to receiving regulatory approval, theoretical concerns have been linked to many OTC switch candidates. These include teratogenic concerns related to adapalene [24], sexual promiscuity concerns related to levonorgestrel [25, 26], and adrenal suppression associated with nasally administered corticosteroids [27]. There is no apparent evidence to suggest that these theoretical concerns have been observed in a nonprescription setting, although the absence of such research does not necessarily confirm there is no risk.

Several countries around the world have made a wide variety of medications, from a diverse range of therapeutic areas, available without a prescription [28]. These observations outline potential opportunities for novel/exceptional OTC switches within the United States, such as medications for acute migraine [29, 30], erectile dysfunction [31, 32], lower urinary tract symptoms in men [33, 34], and antiviral therapy for influenza infection in adults [35]. Beyond these, one could even envision more advanced conditions that often go undertreated as potential targets to address public health concerns. This could include interventions such as rescue therapy for asthma [36]. Technological advances and consumers’ adeptness with technology may make these potentially more complex conditions achievable [37–39]. Simple technology-based solutions, such as web-based self-screening aids that could be accessed on a smartphone along with the expanded space for label information afforded by the interactive interface, may help to facilitate the development of novel OTC drugs. Patient ability to manage their health via technology has been demonstrated extensively with the advent of FDA-cleared medical devices for monitoring heart rate and blood pressure, among other conditions [40–44]. About 10 years ago, the FDA introduced the Nonprescription Safe Use Regulatory Expansion (NSURE) initiative to potentially inspire approaches by sponsors to implement technology to facilitate explorations into more challenging areas [45]. Unfortunately, as evidenced by the present analysis, no advances for consumers have emerged from this initiative.

An evolution to NSURE appears to now be developing in the form of a new proposed rule regarding Additional Conditions for Nonprescription Use (ACNU) from the FDA [46]. The proposed rule outlines many requirements for sponsors and potentially even some disincentives such as dual marketing status for prescription and OTC drugs for the same indication and dose. Under this proposed rule, when labeling alone is not sufficient to ensure that the consumer can appropriately self-select, appropriately use, or both, a sponsor may submit an application proposing an ACNU [46]. While the concept of welcoming more switches via a technology-based approach appears attractive, caution should be exercised pertaining to this idea. If requirements for consumers are substantial and onerous, the technology may prove to produce some of the same inequities that are currently seen in the US healthcare system [47–49].

OTC drugs provide significant benefits in terms of direct and indirect cost-effectiveness for populations that are frequently underserved by the United States healthcare system [50–52]. These groups include people of color, those with lower socioeconomic status, people living in rural areas, and those with no medical insurance or insufficient insurance. If novel OTC drugs are to be successful, they need to serve these groups. To do so, access requirements should be limited rather than burdensome. Bringing a new OTC drug to market with unnecessarily complex requirements will almost certainly not help to address public health concerns and could potentially do more harm than good, particularly from public relations and policy viewpoints. Efforts should be made to mitigate concerns rather than determining success only when all potential issues are fully eliminated. Creating insurmountable obstacles or burdensome restrictions is effectively a decision to leave the status quo in place. Overall benefits and risks in the intended treatment population should be viewed at a macroscopic level, otherwise there are sure to be many missed opportunities to bring solutions to the marketplace. This issue is illustrated with the case of nonprescription statin therapy, where population level benefits appear to outweigh the potential risks [17, 53, 54]. To date, few high impact RTO switches have even made it far enough in the development process to reach an advisory committee such that all interested stakeholder groups can participate in the process.

The primary limitation of this study is that only products included in the FDA RTO switch list were considered. Direct-to-OTC products, devices, and any other consumer medical product were not considered. The data are also limited to approximately the last 20 years. Details before this point are limited but could add perspective.

## Conclusion

In conclusion, while there is great potential for novel OTC drugs to help support consumer autonomy and meaningfully advance public health, particularly for undertreated conditions, relatively few drugs that stem from prescription products have been approved over the last two decades. Many of them have simply followed previously existing products and/or conditions. Despite over 40 RTO switches occurring within the past 20 years, exceptional advancements that expand access to new to OTC active ingredients for new indications remain rare. The underlying practical and policy challenges inhibiting more switches from coming to the market should be investigated.

## Supplementary Information

Below is the link to the electronic supplementary material.

Supplementary file1 (DOCX 18 KB)

## Data Availability

The data that support the findings of this study are available in the FDA Prescription to Over-the-Counter Switch List at https://www.fda.gov/about-fda/center-drug-evaluation-and-research-cder/prescription-over-counter-otc-switch-list.
