# Supplementary material for: A Detailed Analysis of the Past 20 Years of US FDA-Approved Prescription to Over-the-Counter Switches
Source: Ther Innov Regul Sci. 2023 Jun 25;57(5):1074–80. doi: 10.1007/s43441-023-00547-9 (PMC10400472; doi:10.1007/s43441-023-00547-9)
Supplement: Supplementary file 1 — Supplementary file1 (DOCX 18 KB) [file 43441_2023_547_MOESM1_ESM.docx]

**Supplemental material**

Rx-to-OTC Switch List (2002 – 2022) Adapted from the FDA Switch Database

| Year | Brand | Generic | Pharmacological Class |
| --- | --- | --- | --- |
| 2002 | Claritin® Tablets | Loratadine^+^ | Nonsedating Antihistamine^✝^ |
| 2002 | *Claritin® Reditabs | Loratadine^+^ | Nonsedating Antihistamine |
| 2002 | *Claritin® Solution | Loratadine^+^ | Nonsedating Antihistamine |
| 2002 | *Claritin-D® 24-hour | Loratadine / Pseudoephedrine^+^ | Nonsedating Antihistamine / Decongestant |
| 2002 | *Claritin-D® | Loratadine / Pseudoephedrine^+^ | Nonsedating Antihistamine / Decongestant |
| 2002 | *Mucinex® ER Tablet | Guaifenesin | Expectorant |
| 2002 | *Nicotrol® TD | Nicotine | Nicotine Replacement |
| 2003 | *Claritin® Hives Relief Tablets | Loratadine^+^ | Nonsedating Antihistamine |
| 2003 | *Claritin® Hives Relief Reditabs | Loratadine^+^ | Nonsedating Antihistamine |
| 2003 | *Claritin® Hives Relief Solution | Loratadine^+^ | Nonsedating Antihistamine |
| 2003 | Prilosec® OTC | Omeprazole^++^ | Proton Pump Inhibitor^✝^ |
| 2004 | *Mucinex-D® ER Tablet | Guaifenesin / Pseudoephedrine | Expectorant / Decongestant |
| 2004 | *Mucinex-DM® ER Tablet | Guaifenesin / Dextromethorphan | Expectorant / Antitussive |
| 2006 | *Alaway® | Ketotifen fumarate^+^ | Antihistamine / Mast Cell Stabilizer |
| 2006 | Zaditor® | Ketotifen fumarate^+^ | Antihistamine / Mast Cell Stabilizer |
| 2006 | MiraLax® | Polyethylene glycol 3350^++^ | Osmotic Laxative_‡_ |
| 2006 | Plan B® | Levonorgestrel | Hormone Treatment^✝^ |
| 2006 | Lamisil® Derm Gel | Terbinafine | Benzylamine Antifungal |
| 2007 | *Zyrtec® Allergy and Zyrtec® Hives Relief (tablets) | Cetirizine^+^ | Nonsedating Antihistamine |
| 2007 | *Children’s Zyrtec® Allergy and Children’s Zyrtec® Hives (chewable tablets) | Cetirizine^+^ | Nonsedating Antihistamine |
| 2007 | *Children’s Zyrtec® Allergy and Children’s Zyrtec® Hives (solution) | Cetirizine^+^ | Nonsedating Antihistamine |
| 2007 | *Zyrtec-D® | Cetirizine / Pseudoephedrine^+^ | Nonsedating Antihistamine / Decongestant |
| 2007 | Alli® | Orlistat^++^ | Enzyme Blocker^‡^ |
| 2009 | *Zegerid® OTC | Omeprazole-sodium bicarbonate^++^ | Proton Pump Inhibitor |
| 2009 | *Prevacid® 24 HR | Lansoprazole^++^ | Proton Pump Inhibitor |
| 2011 | *Allegra® | Fexofenadine^+^ | Nonsedating Antihistamine |
| 2011 | *Allegra® 24 hr | Fexofenadine^+^ | Nonsedating Antihistamine |
| 2011 | *Allegra-D® 12 hr | Fexofenadine / Pseudoephedrine^+^ | Nonsedating Antihistamine / Decongestant |
| 2013 | *Nasacort® Allergy 24HR (nasal spray) | Triamcinolone acetonide^+^ | Corticosteroid^‡^ |
| 2013 | Oxytrol for Women® | Oxybutynin | Muscarinic Antagonist^✝^ |
| 2014 | *Flonase® Allergy Relief | Fluticasone propionate^+^ | Corticosteroid |
| 2014 | *Nexium® 24 HR | Esomeprazole magnesium^++^ | Proton Pump Inhibitor |
| 2015 | *Rhinocort® Allergy Spray | Budesonide^+^ | Corticosteroid |
| 2016 | *Differin® Gel 0.1% | Adapalene | Retinoid^‡^ |
| 2016 | *Flonase® Sensimist Allergy Relief | Fluticasone furoate^+^ | Corticosteroid |
| 2017 | *Xyzal® Allergy 24HR solution | Levocetirizine dihydrochloride^+^ | Nonsedating Antihistamine |
| 2017 | *Xyzal® Allergy 24HR tablets | Levocetirizine dihydrochloride^+^ | Nonsedating Antihistamine |
| 2020 | *Pataday® Once Daily Relief 0.2% | Olopatadine hydrochloride^+^ | Antihistamine / Mast Cell Stabilizer |
| 2020 | *Pataday® Once Daily Relief 0.1% | Olopatadine hydrochloride^+^ | Antihistamine / Mast Cell Stabilizer |
| 2020 | *Voltaren® Arthritis Pain | Diclofenac sodium | Nonsteroidal Anti-Inflammatory Drug |
| 2020 | *Pataday® Once Daily Relief 0.7% | Olopatadine hydrochloride^+^ | Mast Cell Stabilizer |
| 2020 | *Sklice® lotion, 0.5% | Ivermectin | Anthelmintic^‡^ |
| 2021 | *Astepro® Allergy and Children’s Astepro® Allergy nasal spray, 0.15% | Azelastine hydrochloride^+^ | Antihistamine |
| 2021 | *Lastacaft®, 0.25% | Alcaftadine ophthalmic solution^+^ | Antihistamine |
| 2022 | *Nasonex® 24HR Allergy nasal spray, 50 mcg/spray, metered | Mometasone furoate^+^ | Corticosteroid |

Footnote:

(*) = Notates follow-on drugs

(^✝^) = Notates systemically acting new pharmacological class (at the time of approval)

(‡) = Notates non-systemically acting new pharmacological class (at the time of approval)

(+) = Notates seasonal allergy product

(++) = Notates gastrointestinal product
